# Affordance-based robot manipulation with flow matching

**DOI:** 10.3389/frobt.2026.1906861

**Published:** 2026-09-22

**Authors:** Fan Zhang, Michael Gienger

**Affiliations:** Honda Research Institute EU, Offenbach, Germany

**Keywords:** affordance learning, flow matching, human-centered robotics, robot manipulation, visual learning

## Abstract

We present a framework for assistive robot manipulation that addresses two fundamental challenges: efficient adaptation of large-scale models for scene affordance understanding and effective learning of robot actions by grounding the visual affordance. To tackle the first challenge, we adopt a parameter-efficient prompt tuning method, prepending learnable text prompts to a frozen vision model to predict affordances, while considering spatial and semantic relationships in multi-task scenarios. For the second challenge, we propose a flow matching method, representing a robot visuomotor policy as a conditional process of flowing random waypoints to desired robot actions. We introduce a real-world dataset with 10 tasks to evaluate our approach. Experiments show our prompt tuning method achieves competitive or superior performance to other finetuning protocols across data scales, while satisfying parameter efficiency. Furthermore, flow matching yields more stable training and faster inference compared to diffusion policy; specifically, 1-step flow matching achieves comparable accuracy to 16-step DDIM while reducing inference time by roughly 85%. Our framework seamlessly unifies high-level parameter-efficient affordance representation learning with low-level flow matching policies, illustrating how explicit affordances serve as effective spatial grounding for flow-based policies. https://github.com/HRI-EU/flow_matching.

## 1 Introduction

Recent advances in vision-language models (VLMs) present unprecedented opportunities to solve robot manipulation problems. Attempts in the field have focused on three primary aspects: 1) End-to-end learning manipulation from scratch. These approaches (Padalkar et al., 2023) make the fewest assumptions on tasks and are formulated in language-image-to-action prediction models. 2) Off-the-shelf vision-language models for robot manipulation. These works have prompted pre-trained VLMs in various contexts of robot motion learning, including reward design for reinforcement learning (Ma et al., 2023), python coding (Liang et al., 2023), joint actions (Wang et al., 2023), *etc.*, 3) Intermediate substrate to bridge high-level language-image instructions and low-level robot policies. These works usually introduce some form of prior derived from human knowledge as an intermediate stage to alleviate the sample inefficiency problem of end-to-end learning, including affordances (Huang et al., 2023), primitive skills (Ingelhag et al., 2024), etc. In this paper, we follow the third line of work to unify a parameter-efficient affordance model and a low-level robot flow matching policy.

Extracting affordance knowledge has long inspired the robot community (Xiao et al., 2022). Recent state-of-the-art works have proposed affordance-based robot policies by describing affordances with 6D poses or 2D point tracks (Nasiriany et al., 2024; Bharadhwaj et al., 2024). For example (Nasiriany et al., 2024), represents affordances as the relative poses between the robot end effector and the object at key stages of the task (Bharadhwaj et al., 2024). uses 2D keypoint track predictions to infer a sequence of rigid transforms of the object affordance to be manipulated. Other research decouples affordance learning and robot policy by using more straightforward affordance representations like 2D masks or keypoints (Stone et al., 2023; Yuan et al., 2024). These works typically localize task-specific affordances directly on objects relevant to certain actions, given detailed text instructions. Our affordance model additionally considers the relational context needed for interactions, including the spatial and semantic functional interplay relationship between multiple elements. We concentrate on multi-task scenarios with text prompting. As shown in Figure 1, given the same visual scene but with different language instructions, we aim to extract different affordances for robot policy learning through our proposed model. For example, given a simple instruction of ‘feeding the human’, a sequence of 2D affordance heatmaps on a spoon handle, food, and a person’s mouth are identified.

**FIGURE 1:**
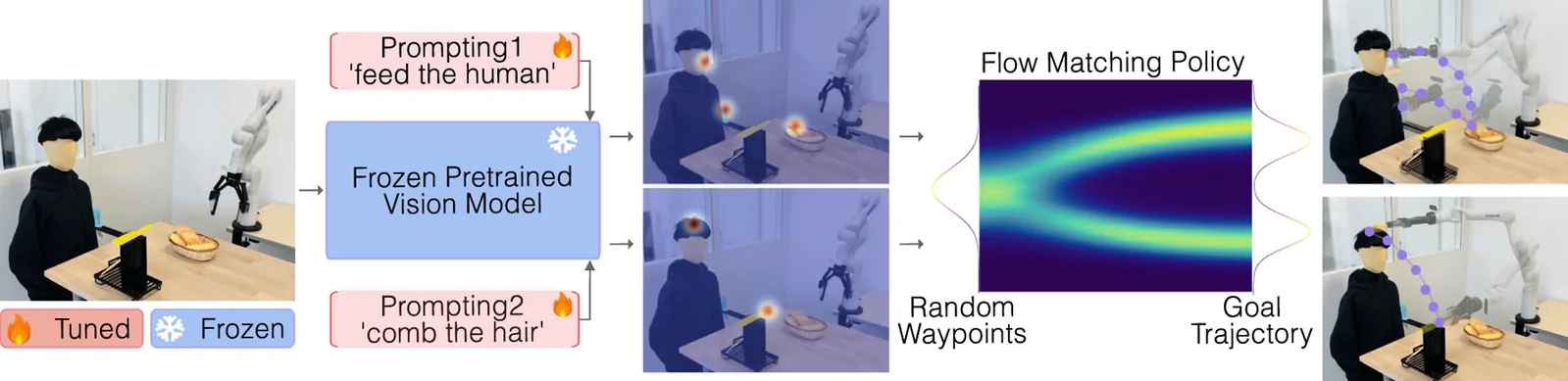
The proposed framework of unifying affordance map learning and action generation for robot manipulation. Given the same visual scene with different language instructions, the model first extracts instruction-relevant manipulation affordances. This is achieved through a prompt tuning method that prepends learnable text-conditioned prompts in a frozen vision foundation model. Then, a flow matching policy is proposed to transform the random waypoints to the desired action trajectories, guided by task-relevant affordance maps.

Recent affordance models have successfully fine-tuned pre-trained vision-language foundation models to extract affordance knowledge (Qian et al., 2024; Yuan et al., 2024). To leverage the capabilities of pre-trained foundation models while simultaneously mitigating the associated computational costs, some works have explored parameter-efficient fine-tuning (PEFT) large vision-language models (Jia et al., 2022). One representative line of PEFT research has concentrated on prompt tuning methods, which prepend learnable prompts to a large frozen pre-trained model and optimize them via gradients during finetuning. In contrast to studies in NLP, it has been shown that prompt tuning could match or even outperform full fine-tuning and adapter-based methods, but with substantially less parameter storage in visual domains (Zhu et al., 2023). Inspired in part by the notion of human cognitive penetrability mechanism (Maier and Abdel Rahman, 2019) that uses linguistic knowledge to tune ongoing visual processing, we aim to incorporate learnable text-conditioned prompts into any vision foundation model while keeping it frozen, preserving its visual understanding capabilities, to learn instruction-relevant manipulation affordance maps.

The subsequent challenge involves deploying the visual affordance across robot manipulation learning paradigms. A recent line of work builds on successes in curriculum-based imitation learning (Blessing et al., 2023), probabilistic movement primitive modeling (Yildirim and Ugur, 2024), and diffusion models (Chi et al., 2023) to generate motion trajectories to capture multimodal action distributions. Flow Matching is another novel generative method. Sharing theoretical similarities with the diffusion process, flow matching aims to regress onto a deterministic vector field to flow samples toward the target distribution (Lipman et al., 2022). has proven that the simplicity of flow matching objectives allows enhanced performance in stable training and generation quality compared to solving complex stochastic differential equations in the stochastic denoising diffusion process. We extend flow matching to the robotics domain. As shown in Figure 1, the proposed method would flow the random waypoints to the desired action trajectories based on multi-task affordances in a single flow matching policy.

We also construct a Real-world Activities of Daily Living (ADLs) dataset with 10 tasks. The novelty of our dataset is that it contains the same scenarios but with multi-task affordance and robot trajectories. Experimental evaluation on our dataset empirically demonstrates that the prompt tuning method for learning affordances achieves performance competitive with, and sometimes beyond, other finetuning protocols across data scales and vision-language fusion architectures.

This work seamlessly grounds high-level task understanding and fine-grained low-level motor control for real-world robot manipulation. Our framework establishes a synergistic link between prompt-tuned affordance learning and flow-matching policy. Rather than treating these as decoupled modules, we leverage learned affordance maps as explicit spatial-semantic conditioners for the flow-matching policy. Our parameter-efficient prompt tuning extracts dense affordance representations that identify where and how an object should be interacted with based on task intent (e.g., locating the handle vs. the bristle tip in the task of ‘brushing the teeth’). We leverage the capability of the flow matching policy to represent multi-modal action distributions for learning robot actions, while affordance maps readily provide sufficient guidance in shaping the policy. We have further systematically evaluated robot manipulation with flow matching across several benchmarks, encompassing various input representations, robot control types, and manipulation tasks—including 3D trajectory prediction on our dataset as well as 6D manipulation on public benchmarks. We showcase that across several benchmarks, flow matching attains enhanced performance in training stability and computational efficiency amongst competing methods of behavior cloning. By coupling semantic affordance conditioning with flow matching’s continuous trajectory generation, our unified framework achieves both flexible multi-task understanding and precise manipulation. Our code is publicly available.

The main contributions can be summarized as follows:

1. Parameter-Efficient Affordance Learning: A parameter-efficient prompt tuning method for adapting pretrained vision foundation models conditioned on language instructions to learn manipulation affordances, incorporating the spatial and semantic interactions between elements in multi-task scenarios. We contribute an ADLs dataset containing the same scenarios but with multi-task affordance annotations and trajectory ground truths to validate our proposed affordance learning method.
2. Principled Advancements in Policy Learning: A novel formulation using flow matching for robot manipulation learning from various inputs, including visual affordances. Empirical and extensive results on our dataset and public benchmarks show that flow matching leads to consistently enhanced results compared to some alternative behavior cloning methods in various manipulation tasks. This includes more stable training and evaluation and noticeably faster inference, while maintaining comparable generalization performance to diffusion policies (DDIM, DDPM, and score-based models).
3. Synergistic Modular Architecture for Manipulation: A framework seamlessly unifies parameter-efficient affordance learning and robot action generation with flow matching. Experimental results, compared against end-to-end learning and off-the-shelf VLM-based trajectory generation frameworks, demonstrate that explicit spatial affordances provide key structural guidance to shape flow matching policies for robot manipulation.

## 2 Related work

### 2.1 Affordance learning and affordance-based robot policy

Humans rely heavily on affordances to perform day-to-day tasks across environments efficiently. The concept of affordance has been introduced in (Gibson, 2014), referring to the ability to perform certain actions with objects in the context of a given scene. Several state-of-the-art works have adopted this concept and successfully proposed affordance-based robot policies by learning the affordance parameterization that could be amenable to deployment on robots, including 6D pose (Nasiriany et al., 2024), stable object placement (Zeng et al., 2021), point tracks (Bharadhwaj et al., 2024), and post-contact trajectory (Bahl et al., 2023). Other approaches decouple actionable representation for affordances from robot policy by using more straightforward affordance representations, like 2D masks (Stone et al., 2023), keypoints (Manuelli et al., 2019), heatmaps (Zhang and Demiris, 2022), part segmentation (Do et al., 2018), dense image feature descriptors (Florence et al., 2018). We follow this idea of decoupling and use 2D heatmaps as affordances.

Recent SoA works have successfully leveraged pre-trained vision-language models (e.g., LLaVA and Vicuna) to extract affordance knowledge, as in AffordanceLLM (Qian et al., 2024) and Robopoint (Yuan et al., 2024). This raises three important research questions: i) How can language-vision inputs for instruction-aware vision encoding be readily fused? ii) How to parameter-efficiently finetune a pretrained foundation model? and iii) How to encode the spatial and semantic functional interplay relationship between elements in multi-task scenarios for robot manipulation affordance learning? We introduce a prompt tuning method as a solution to these questions.

### 2.2 Parameter-efficient finetuning

Given the dominance of large-scale vision-language models, many approaches have been proposed to efficiently finetune a frozen pretrained model for downstream tasks to speed up training and reduce memory (Gupta et al., 2022; Radford et al., 2021). Two representative parameter-efficient finetuning methods are adapters and prompting. The adapter-based research designs adapter variants that could add extra lightweight modules (Gao et al., 2024; Hu et al., 2021; Liu et al., 2023b; Sharma et al., 2023). Other work focuses on prompt tuning (Liu et al., 2021; Liu et al., 2023a), which treats learnable prompts as continuous vectors and computes their gradients with backpropagation during training. Studies on randomly generalised trainable prompts (Jia et al., 2022) for universal use or condition-admitted prompt variables (Sohn et al., 2023) for better specific task performance have both been explored. The extension of prompt tuning to vision tasks has gained massive success. Visual prompt tuning (Jia et al., 2022) has manipulated visual prompts to steer models in arbitrary vision tasks. Dancemvp (Zhong and Demiris, 2024; Zhong et al., 2023) has adjusted language prompts to guide text-audio models in performing dancing assessment tasks. Inspired by its recent success, we extend the prompt tuning technologies to address the challenge of adapting large pretrained vision-language models to affordance learning for robot manipulation. The intuition is clear: if the model understands the posed text instruction and the inherent context, it should extract visual affordances that directly correspond to the relevant image aspects. Our method achieves the above goal by integrating learnable text-conditioned prompts into a large vision encoder, while keeping it frozen to preserve visual understanding capabilities. Besides, recent research on VLM for affordance-based robot policy typically localizes task-specific affordances directly on objects relevant to certain actions, which requires detailed language instructions about all task-related objects and actions. We aim to enable our affordance model to incorporate relational context essential for interactions, capturing both spatial and semantic-functional relationships among multiple objects and persons, where only simple prompts are required.

### 2.3 Robot learning from demonstration

Imitation learning has been a common paradigm for robots, which requires simulated or real-world demonstration data collection (Levine et al., 2016; Mirza and Singh, 2025). To improve data efficiency, extensive work has been proposed to learn robot policies on top of visual representations (Liu et al., 2024), such as keypoints or affordance heatmaps (Liu et al., 2024), instead of end-to-end raw images (Goyal et al., 2023). This paper concentrates on using affordances to guide low-level robot manipulation. In terms of network architectures for robot learning, prior works have successfully investigated convolutional networks (Levine et al., 2016), Transformers (Sridhar et al., 2023), generative adversarial networks (Ho and Ermon, 2016), Energy-Based Models (Florence et al., 2022), etc. However, the collected data is usually expected to be non-convex and multi-modal due to the variability in human demonstrations. Recent works have addressed this problem by reformulating the robot policy as a generative process. Diffusion policy (Chi et al., 2023), including Denoising Diffusion Probabilistic Models (DDPM (Ho et al., 2020)), Denoising Diffusion Implicit Models (DDIM (Song et al., 2020a)) and score-based models (Reuss et al., 2023), has emerged as a powerful class of generative models for behavior cloning by representing a robot’s visuomotor policy as a conditional denoising diffusion process. In this work, we investigate flow matching (Lipman et al., 2022), a novel generative model that has demonstrated its superiority in image generation but is much less explored in robotics domains.

### 2.4 Flow matching in robotics

Despite its recent progress in image generation (Albergo and Vanden-Eijnden, 2022), the application of flow matching in robotics domains remains underexplored (Hu et al., 2024; Rouxel et al., 2024). Few prior studies have concentrated exclusively on certain robot scenarios for deploying flow matching, for pointcloud environments (Chisari et al., 2024), dynamics (Nguyen et al., 2025), Riemannian manifolds (Braun et al., 2024), etc. We propose to use flow matching to learn multi-task robot behaviors from raw observations, including visual affordances obtained from a vision-language model, in a single supervised policy. We have first systematically evaluated robot manipulation with flow matching on several benchmarks, including various input representations, robot control types, and manipulation tasks.

## 3 Materials and methods

### 3.1 Prompt tuning for affordance map learning

Given any type of pre-trained vision transformer, our objective is to learn a set of text-conditioned prompts to maximize the likelihood of correct affordance labels, as shown in Figure 2. Only the prompt-related layers and the decoder are being updated during training, while the vision transformer remains frozen. Inspired by Vision Prompt Tuning (Jia et al., 2022), we propose two frameworks: shallow and deep network architectures.

**FIGURE 2:**
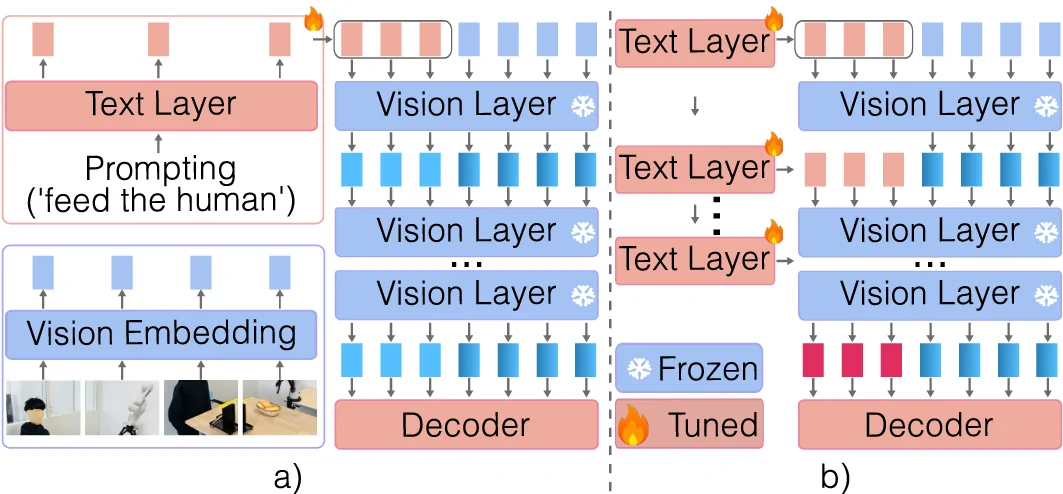
Overview of prompt tuning structures used for affordance learning. (Left) For the shallow structure, text-conditioned prompts are prepended to the first vision transformer layer. (Right) For the deep structure, prompts are inserted into every vision layer. Only the prompt-related layers and the decoder are being updated during the training, while the vision transformer remains frozen. **(a)** shallow architecture, **(b)** deep architecture.

#### 3.1.1 Shallow architecture

The vision transformer layer takes the image patch embeddings $E_{0}$ as input and passes through various layers $L_{i}^{v}$ to achieve vision features $E_{i}$ , where $E_{i}\in R^{M\times C}$ and C is the channel dimension. This can be described as:

$$E_{i}=L_{i}^{v}\left(E_{i-1}\right)\;i=1,2,\ldots ,N$$

Similarly, the text transformer layer could be represented as

$$P_{i}=L_{i}^{p}\left(P_{i-1}\right)\;i=1,2,\ldots ,N$$

where $P_{0}$ denotes the text tokens, text features $P_{i}$ are obtained through various layers $L_{i}^{P}$ , where $p_{i}\in R^{K\times C}$ .

As shown in Figure 2, for the shallow structure, only one text transformer layer is used to compute text features $P_{1}$ , which are then treated as prompts and inserted into the first vision transformer Layer:

$$\begin{array}{ll} \left[Z_{1},E_{1}\right] & =L_{1}^{v}\left(\left[P_{1},E_{0}\right]\right)\\ \left[Z_{i},E_{i}\right] & =L_{i}^{v}\left(\left[Z_{i-1},E_{i-1}\right]\right) \end{array}$$

Then a decoder is added on the global output flattened token sequence to generate visual affordance tokens.

$$\text{Affordance}=\text{Decoder}\left(Z_{N},E_{N}\right)$$

#### 3.1.2 Deep architecture

For the deep architecture, the only difference is that text features $P_{i}$ are computed through each layer and introduced at the corresponding vision transformer layer’s input space:

$$\begin{array}{ll} \left[\text{\_},E_{1}\right] & =L_{1}^{v}\left(\left[P_{1},E_{0}\right]\right)\\ \left[\text{\_},E_{i}\right] & =L_{i}^{v}\left(\left[P_{i},E_{i-1}\right]\right) \end{array}$$

This is different from parallel adapters, which introduce additional trainable modules that run in parallel with the main transformer layers instead of modifying existing layers.

#### 3.1.3 Implementation details

In our implementation, we select and adopt a single structure from the two designs. The deep structure demonstrates better performance than the shallow structure, albeit with increased computational cost. Our goal is to integrate textual representations into any vision encoder while keeping it frozen, preserving its visual understanding capabilities. Thus, we have chosen the most basic vision backbone, a pretrained ViT-B-16 transformer. We use the classic CLIP Transformer layers (Radford et al., 2021) that output 76 tokens. As suggested by MAE (He et al., 2022), the decoder is only used for downstream tasks and could be flexible and lightweight. Thus, we use a single transformer decoder layer.

We use the L2 Mean Squared loss between the predicted and ground truth affordances for network training. The training parameters for the prompt tuning network include an image size of 224 × 224, AdamW with a learning rate of 1.5e-4, including Warmup with step-decay, and a batch size of 256. We add positional embeddings to all the image and language tokens to preserve the positional information. In the subsequent experiments, we will further ablate multiple model variants, including text and vision fusion structures, prompt depth, pretrained weights for vision transformers, etc.

### 3.2 Flow matching policy

We build the robot behavioral cloning policy as a generative process of flow matching, which constructs a flow vector that continuously transforms a source probability distribution toward a destination distribution. Flow matching leverages an ordinary differential equation to deterministically mold the data distribution, contrasting with Denoising Diffusion Probabilistic Models (DDPM), which are based on a stochastic differential equation by introducing noise.

#### 3.2.1 Flow matching model

Given a conditional probability density path $p_{t}\left(x|z\right)$ and a corresponding conditional vector field $u_{t}\left(x|z\right)$ , the objective loss of flow matching could be described as:

$$L_{\text{FM}}\left(\theta \right)=E_{t,q\left(z\right),p_{t}\left(x|z\right)}{\left\|v_{t}\left(x,\theta \right)-u_{t}\left(x|z\right)\right\|}^{2}$$ (1)

where $x∼p_{t}\left(x|z\right)$ , t∼U[0,1] . Flow matching aims to regress $u_{t}\left(x|z\right)$ with a time-dependent vector field of flow $v_{t}\left(x,\theta \right)$ parameterized as a neural network with weights θ . $u_{t}\left(x|z\right)$ can be further simplified as:

$$u_{t}\left(x|z\right)=x_{1}-x_{0}\;x_{0}∼p_{0},x_{1}∼p_{1}$$

$p_{0}$ represents a simple base density at time t=0 , $p_{1}$ denotes the target complicated distribution at time t=1 , $x_{0}$ and $x_{1}$ are the corresponding samplings. $v_{t}\left(x,\theta \right)$ is described as:

$$v_{t}\left(x,\theta \right)=v_{\theta }\left(x_{t},t\right)$$ (2)

where we define $x_{t}$ as the linear interpolation between $x_{0}$ and $x_{1}$ with respect to time $x_{t}=tx_{1}+\left(1-t\right)x_{0}$ , following the linear conditional flow theory (Peyré and Cuturi, 2019). $v_{\theta }$ is a network of the flow model. Thus Equation (Equation 1) could be reformatted as

$$L_{\text{FM}}\left(\theta \right)=E_{t,∼p_{0},∼p_{1}}{\left\|v_{\theta }\left(x_{t},t\right)-\left(x_{1}-x_{0}\right)\right\|}^{2}$$ (3)

where $E_{t∼U\left(0,1\right),\;x_{0}∼p_{0},\;x_{1}∼p_{1}}\left[\cdot \right]$ denotes the expectation evaluated over time t sampled uniformly from [0, 1], initial noise samples $x_{0}$ drawn from the prior distribution $p_{0}$ , and target data samples $x_{1}$ drawn from the empirical target distribution $p_{1}$ . This represents the progression of the scalar flow that transforms data from source to target between time 0 and 1.

#### 3.2.2 Flow matching for visuomotor policy learning

We extend flow matching to learn robot visuomotor policies. This requires two modifications in the formulation: i) modeling the flow estimation conditioned on input observations o ; ii) changing the output x to represent robot actions. Figure 3 illustrates our model structures.

**FIGURE 3:**
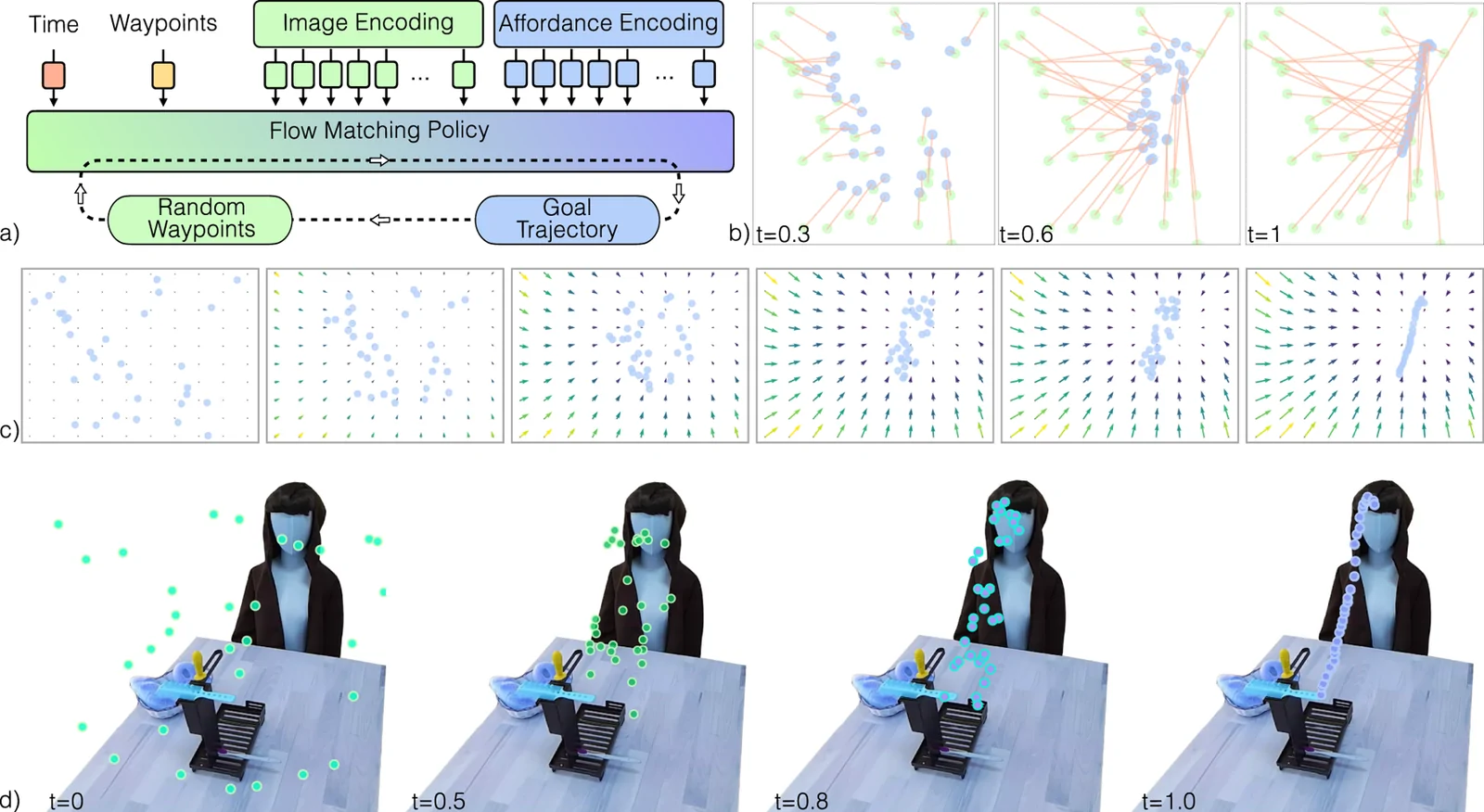
Framework of flow matching policy. **(a)** General formulation. At each time step, flow matching takes visual observation o (e.g., state-based inputs, RGB-D images, visual affordances) as input, and outputs robot actions (e.g., 6D robot end-effector trajectories, robot joint actions, gripper actions). **(b–d)** Visualization of the inference process of transforming random waypoints to target actions over time from 0 (green) to 1 (purple). Red lines in **(b)** denote the flow paths. Vector fields are shown in **(c)**.

Visual observation Conditioning: We modify Equation (Equation 2) to allow the model to predict actions conditioned on observations:

$$v_{t}\left(x|o\right)=v_{\theta }\left(x_{t},t|o\right)$$

Closed-loop action trajectory prediction: We execute the action trajectory prediction obtained by our flow matching model for a fixed duration before replanning. At each step, the policy takes the observation data o as input and predicts Tp steps of actions, of which Ta steps of actions are executed on the robot without replanning. Tp is the action prediction horizon and Ta is the action execution horizon. The whole training process of flow matching is illustrated in Algorithm 1.

Algorithm 1 Robot flow matching policy training.

**Input:** observation o , target robot actions $x_{1}$ , source random waypoints $p_{0}$

**Output:** flow $v_{\theta }$

1: **while** not converged **do**
2: $x_{0}∼p_{0}$ , sample random robot waypoints
3: t∼U[0,1] , sample time steps
4: $x_{t}=tx_{1}+\left(1-t\right)x_{0}$ , linear interpolation
5: $v_{t}\left(x|o\right)=v_{\theta }\left(x_{t},t|o\right)$ , flow estimation
6: $\nabla_{\theta }\left\|v_{\theta }\left(x_{t},t|o\right)-{\dot{x}}_{t}\right\|$ , gradient step
7: **end** **while**
8: Stopping criteria: training epochs reached

Inference: For the inference procedure, random waypoints are sampled from the source distribution and then flowed into the target trajectory by estimating the flow from t=0 to t=1 over steps. We could use multiple steps 1/Δt for inference:

$$x_{t+\Delta t}=x_{t}+\Delta tv_{\theta }\left(x_{t},t|o\right),\;\text{for}\;t\in \left[0,1\right]$$ (4)

#### 3.2.3 Implementation details

For the network structures of flow matching, we first use ResNet (He et al., 2016) for visual embeddings o . The flow model $f_{\theta }$ is represented with U-Net (Ronneberger et al., 2015). The flow model predicts vectors $v_{t}$ conditioned on visual observation embeddings o with Feature-wise Linear Modulation (FiLM) (Perez et al., 2018) as well as the interpolated waypoints $x_{t}$ . In the subsequent experiments, we will further study multiple model variants, including a transformer-based structure and trajectory representation.

In our case of robot manipulation, $x_{1}$ in Equation (Equation 3) represents the demonstration robot action trajectories. $x_{0}$ is the random generated waypoints following a multivariate normal distribution $x_{0}∼N\left(0,I\right)$ . x here could denote 6D robot end-effector trajectories, robot joint actions, gripper actions, etc. The visual embeddings o include various types of inputs, such as state-based inputs, RGB-D images, and visual affordances.

#### 3.2.4 Comparisons against diffusion policy

In this section, we provide some insights and intuitions about flow matching and its comparisons against diffusion policy for clarification. Algorithm 1 and Algorithm 2 respectively show the pseudocode of training flow matching and diffusion policy with DDPM. We can see several similarities and differences between these two methods:

- Sampling: By solving a Stochastic Differential Equation (SDE), DDPM generates a clean sample from Gaussian noise. Flow matching regresses onto a target flow vector field that generates a deterministic mapping from source to target data distributions by solving an ordinary differential equation (ODE), which drops all the Gaussian assumptions.
- Probability path: Flow matching includes a particularly interesting family of probability paths: the conditional vector field with linear interpolant. Flow matching paths with linear interpolation are simpler than diffusion paths, forming straighter trajectories, whereas diffusion paths result in curved paths. These properties seem to empirically translate to more stable training, faster generation, and better performance.
- Reparameterization: (Lu and Song, 2024; Nakkiran et al., 2024) claims that diffusion models and flow matching can be considered equivalent with reparameterization, including rescaling time and reparameterizing the marginal velocity field via the score function. Specifically (Nakkiran et al., 2024), argues that DDIM is equivalent to flow-matching with the following parameters:

$$\begin{array}{lll} v_{t}^{\left[x_{1},x_{0}\right]}\left(x_{t}\right) & ≔\frac{1}{2t}\left(x_{t}-x_{0}\right) & \text{DDIM}\\ v_{t}^{\left[x_{1},x_{0}\right]}\left(x_{t}\right) & ≔\frac{1}{2\sqrt{t}}\left(x_{0}-x_{1}\right) & \text{Flow Matching} \end{array}$$

can both generate the same trajectory:

$$x_{t}=x_{0}+\left(x_{1}-x_{0}\right)\sqrt{t}$$

Algorithm 2 Robot diffusion policy training.

**Input:** observation o , target robot actions $x_{1}$ , source Gaussian noises $p_{0}$

**Output:** noise $\epsilon_{\theta }$

1: **while** not converged **do**
2: $x_{0}∼p_{0}$ , sample Gaussian noises
3: t∼U[0,1] , sample time steps
4: $x_{t}=\sqrt{{\bar{\alpha }}_{t}}x_{1}+\sqrt{1-{\bar{\alpha }}_{t}}x_{0}$ , noise-data interpolation
5: $\epsilon_{t}\left(x|o\right)=\epsilon_{\theta }\left(x_{t},t|o\right)$ , noise estimation
6: $\nabla_{\theta }\|\epsilon_{\theta }\left(x_{t},t|o\right)-x_{0}\|$ , gradient step
7: **end** **while**
8: Stopping criteria: training epochs reached

That being said, DDIM at time t corresponds to flow matching at time $2\sqrt{t}$ ; thus, flow matching is “slower” than DDIM when t is small. This may be beneficial for flow matching in practice.

In conclusion, flow matching is a mathematical generalization of Diffusion (DDPM). The ‘straighter flows’ result from linearly interpolated paths that minimize curvature. This geometric optimization allows ODE solvers to use significantly fewer steps without the high discretization error inherent to the curved SDE paths of DDPM.

### 3.3 Activities of Daily Living dataset

We construct a real-world dataset with 10 tasks across Activities of Daily Living. Each task includes 1, 000 sets of RGB-D images, demonstrated robot action trajectories, and labeled ground truth of affordance maps. Thus, 10, 000 demonstrations have been collected in total. The novelty of our dataset includes: (i) It contains the same scenarios with multiple objects, multi-task affordances, and demonstrated robot trajectories. (ii) All tasks are related to Activities of Daily Living that involve (simulated) human data.

Dataset collection was conducted via kinesthetic teaching of the robot end-effector, with images and trajectories recorded using the Robot Operating System (ROS) at a sampling rate of approximately 30 Hz . For each task, affordance heatmaps were constructed to represent the object regions that physically interact with the robot during execution. Specifically, these ground-truth affordances were annotated using 2D Gaussian distributions centered on the target interaction pixels for demonstrated actions. For example, the feeding task includes affordance distributions over the fork handle, the target food item, and the user’s mouth. To ensure annotation consistency across the dataset, data collection and labeling were conducted by two expert annotators (i.e., the authors).

Objects are randomly placed within the range of table (1.5 m × 1 m). The human (manikin) is placed randomly around the table in the camera view. We have around 30 different objects. Our tasks include prompt primitives: ‘sweep the trash’, ‘pass the water to the human’, ‘hang the towel’, ‘put on the hat’, ‘cover the food’, ‘wipe the nose’, ‘wipe the forearm’, ‘feed the human’, ‘comb the hair’, and ‘brush the teeth’. We can also optionally add a pretrained LLM layer (e.g., GPT) in the very front for zero-shot text classification, allowing for linking other language instructions to one of the ten prompt primitives. For example, an ambiguous instruction ‘I am hungry’ can be linked to the prompt primitive ‘feed the human’. The camera is positioned with some variations but generally oriented towards the table and objects. Please refer to the Supplementary Material for more videos of each task.

## 4 Results

In this section, we systematically evaluate the performance of the proposed prompt tuning and flow matching methods.

In Section 4.1, we first benchmark our proposed prompt-tuning structures against several commonly used parameter-efficient fine-tuning approaches to demonstrate the superior performance of prompt tuning. We then compare our method with state-of-the-art VLM-based affordance learning approaches to highlight its efficiency. Additionally, we perform ablation studies to analyze the impact of different design choices on performance. In Section 4.2 and 4.3, we compare our flow-matching policy with state-of-the-art generative learning, imitation learning, and VLA models for robot manipulation through real-world experiments, evaluating performance in terms of generation quality, stability, inference time, and training resource requirements. In Section 4.4, we systematically investigate the performance of flow matching compared to diffusion policy, across simulation and real-world benchmarks.

### 4.1 Affordance evaluation with prompt tuning

#### 4.1.1 Baseline studies

We benchmark our proposed prompt tuning structures against several commonly used parameter-efficient finetuning protocols and SoA VLM-based affordance learning methods:

- Full fine-tuning: fully update the text and vision transformer layers and the decoder.
- Adapter-based methods: insert MLP layers with residual connections between pretrained frozen transformer layers of vision and language, as customary in the literature (Liu Z. et al., 2023; Sharma et al., 2023).
- Side-network methods: train a language-based network on the side, append pretrained vision features and sidetuned text features before being fed into the decoder, as customary in the literature (Ganz et al., 2024). This also shares similarities with parallel adaptors. Our deep prompt tuning method differs in the sense that it inserts text layers into every vision layer, while parallel network methods introduce additional trainable modules that run in parallel with the main transformer layers instead of modifying existing layers.
- Decoder-based methods: adopt the pretrained backbone as a feature extractor with fixed weights during tuning, and only train the decoder, as customary in the literature (He et al., 2016).
- Cross-attention methods: use cross-attention fusing text and vision instead of simple prepending. An example of cross-attention fusing vision and language can be found in the literature (Jiang et al., 2022).
- Mixed-design methods: mix the favorable adaptor designs. We compare against the state-of-the-art MAM adaptor (He et al., 2021), which is a Mix-And-Match adapter designed based on the practical findings on NLP.
- VLM-based affordance learning: We also compare against two state-of-the-art affordance learning methods based on fine-tuning large VLMs: AffordanceLLM (Qian et al., 2024) and Robopoint (Yuan et al., 2024). Both AffordanceLLM and Robopoint were fine-tuned on our dataset to ensure a fair comparison. Because Robopoint predicts action keypoints directly, it does not generate intermediate affordance heatmaps, which accounts for its absence in our table.

For a fair comparison, all the baselines here use self-supervised pretrained MAE weights on ImageNet-21k dataset for the vision transformer model. We randomly split our Real-world Activities of Daily Living (ADLs) dataset into 80%–20% for training and testing. The results reported here are obtained after 1, 000 epochs of training.

#### 4.1.2 Main results

We use two metrics to evaluate our results: (i) L2 error of affordance heatmap estimation, and (ii) L2 distance between the predicted and ground truth of heatmap centers. We fit Gaussian Mixture Models on predicted heatmaps to determine the inferred heatmap centers. The heatmap error is averaged on each map, and the center error is averaged on each center point. Three observations could be made:

- General analysis: Table 1 presents the results of prompt tuning on our ADLs testing dataset for affordance learning, comparing against baselines. The deep structure of prompt tuning outperforms other parameter-efficient baselines. We can also see that deep prompt tuning achieves better performance than RoboPoint but falls short of the performance achieved by AffordanceLLM. We would like to mention that AffordanceLLM and Robopoint are not parameter-efficient models (the focus of our research), as they involve finetuning large models, including LLaVA and Vicuna, on our dataset. Full finetuning such a large model may not function optimally if only a small dataset is available.
- Prompt tuning against full finetuning: Full finetuning slightly outperforms deep prompt tuning in terms of heatmap estimation error and heatmap center error. However, the distinction of heatmap center errors (1.78 pixels) remains subtle, given the full image size of 224×224 . This outcome indicates that most heatmap errors are caused by the tails of the Gaussian distribution, instead of the center area where the robot actions are actually applied. We will further ablate the impact of dataset size on these two methods.
- Generalizability: We also observe that the trained model could be generalized to new objects. For example, the training dataset only includes a manikin. We found out that it generates well on our testing data with real humans. Affordances on objects with similar shapes (e.g., forks and spoons) could also be transferred. Note that as the proposed tuning method is parameter-efficient, it is envisaged that the method could be readily transferred to different tasks with a small amount of task-specific data. Note that the testing experiments involved only the authors as participants and were therefore exempt from the requirement for ethics approval.
- Prompt tuning or adapters: As pointed by the research of Visual Prompt Tuning (Jia et al., 2022), in contrast to comparable studies in NLP, prompt tuning outperforms full fine-tuning and adapter-based methods in the visual domain. The MAM adaptor (He et al., 2021) mixes the favorable adaptor designs based on the practical findings on NLP and achieves state-of-the-art results, but does not function optimally in the image-text domain.

**TABLE 1:** Results of prompt tuning baseline and ablation studies.

Category	Methods	Learnable params ↓	Affordance heatmaps $\left(\times 10^{-3}\right)$ ↓	Heatmap centers (pixel) ↓
Baselines	Full	153.8M	0.76	1.15
	Decoder	3.9M	1.51	13.48
	Adapter	19.2M	1.17	6.22
	Cross-attention	43.5M	1.26	8.89
	Side-network	42.7M	1.35	9.20
	Mixed-design	108M	0.85	2.96
	AffordanceLLM	7B	0.72	1.01
	Robopoint	12B	-	3.18
Ours	PT-shallow	8.0M	1.42	12.04
	PT-deep (self-supervised weights)	42.1M	0.80	2.93
Ablations	PT-deep (supervised weights)	42.1M	1.48	10.13
	PT-deep (image output)	42.1M	1.56	13.27

We report the number of learnable parameters, the heatmap estimation error (the fourth column) and the heatmap center error (the fifth column). Our method achieves state-of-the-art results among parameter-efficient techniques, narrowing the performance gap to full fine-tuning and stronger foundation models. Bold values indicate the best results achieved by our proposed method (distinguished from full fine-tuning and foundation models that require significantly more parameters).

What do prompts learn? We show a t-SNE (Van der Maaten and Hinton, 2008) visualization of the embeddings after the last vision transformer layer (before the decoder) in Figure 4. We can see that the points of the same color (e.g., tasks with the same language prompts) are embedded together, implying that the representations recover the underlying manifold structure of discriminative task information. We also observe that our efficient prompt tuning model is capable of learning joint spatial and semantic dependencies in multi-task ADL scenarios, considering tasks involving objects with multiple interaction zones.

- Semantic Disambiguation: When given task prompts like *‘brush the teeth’* versus *‘comb the hair’*, task-specific prompt tuning steers the attention mechanisms of the frozen vision foundation model. This dynamically isolates distinct semantic targets, focusing narrow attention on the toothbrush handle or hairbrush grip for grasping, while semantically grounding the target affordance zone on the human face or head.
- Spatial Relationship Grounding: By coupling affordances with our flow-matching policy, our method does more than just select visual regions; it actively encodes relational spatial contexts. Together, this integrated framework internally incorporates local object geometry alongside inter-object spatial arrangements (e.g., relative approach directions and alignment angles between the end-effector and target human contours), ultimately conditioning the policy to generate precise, context-aware task trajectories.

**FIGURE 4:**
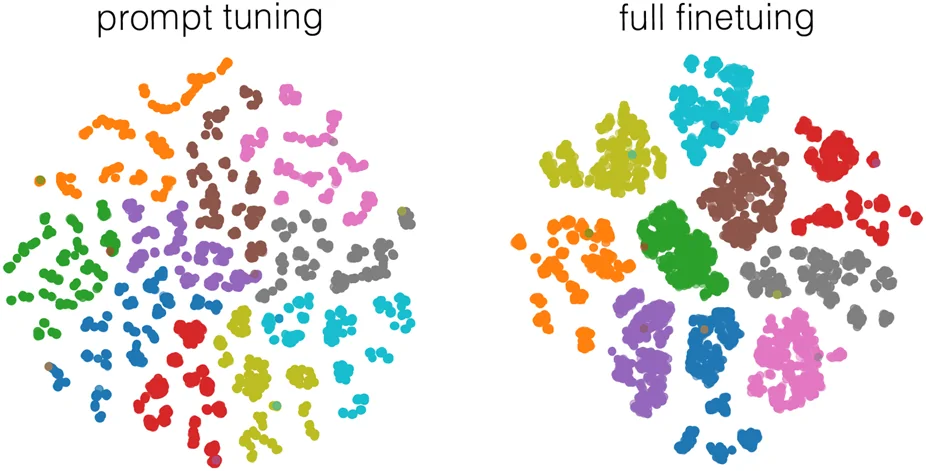
t-SNE visualizations of the embeddings before the decoder. The points of the same color denote the tasks with the same language prompts, which are embedded together. The prompt tuning method could produce instruction-relevant features without updating vision backbone parameters.

#### 4.1.3 Ablations

We further ablate model design choices:

Pretrained Weights: We evaluate using MAE self-supervised pretrained weights and supervised pretrained weights trained on ImageNet-21k dataset for the vision transformer model. The results in Table 1 show that self-supervised pretrained weights perform better. We are aware of other more complicated variants of vision transformers, for example, CLIP vision encoder and its pretrained weights. As our goal is to integrate textual representations into any vision encoder while keeping it frozen, we have chosen the most basic ViT-B-16 transformer backbone and commonly used pretrained weights and achieved competitive results.

Decoder Input: We apply the decoder on the global output and image-corresponding output after the vision transformer respectively, and report results in Table 1.

Dataset Size: We use various amounts of data for training. Figure 5-left shows that prompt tuning has better adaptability than full finetuning when downstream data is scarce.

**FIGURE 5:**
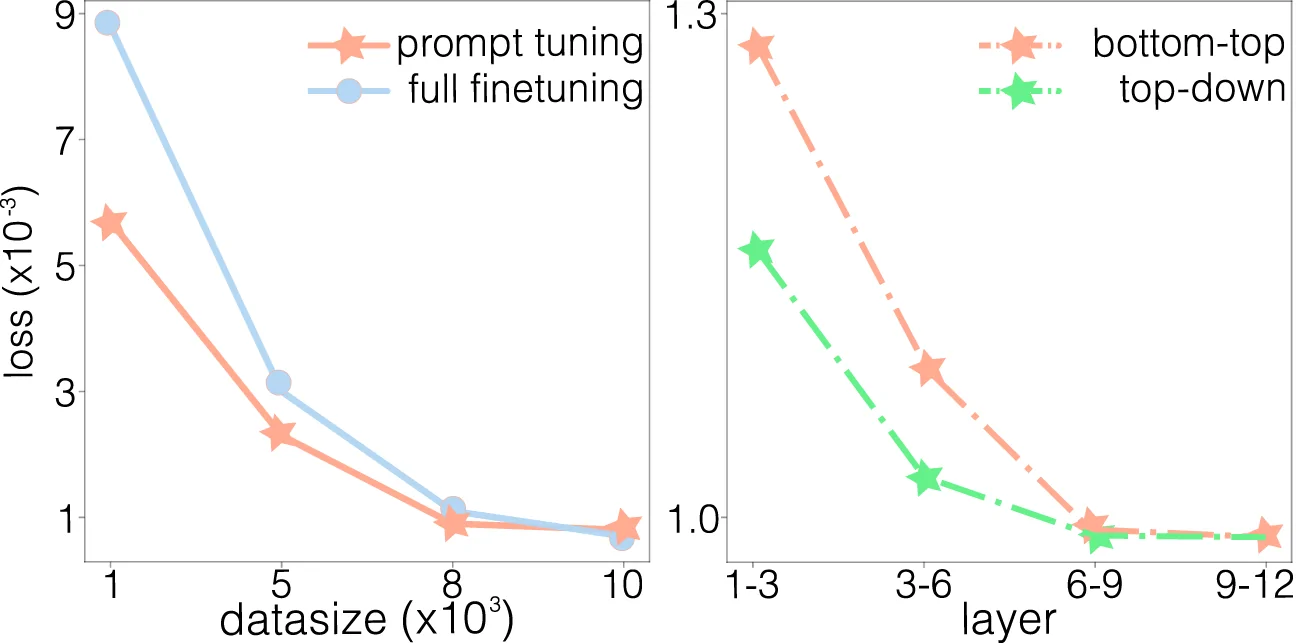
Ablation studies of prompt tuning. We investigate the effect of various design choices on affordance learning performance, including pretrained weights, decoder input, dataset size, and prompt location.

**FIGURE 6:**
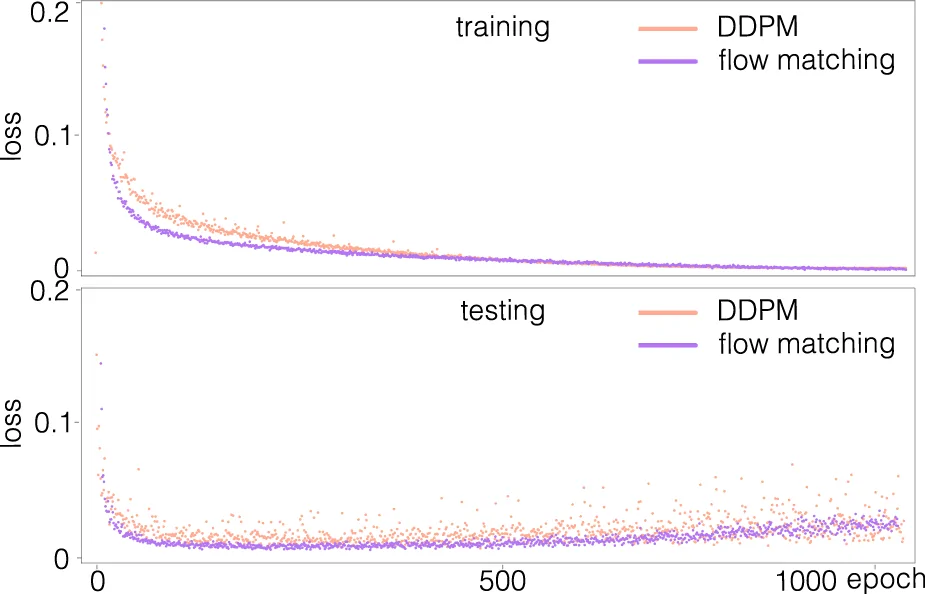
Training and testing loss throughout the training process. Flow matching exhibits greater stability on both training and evaluation than diffusion policy.

Prompt Location: We have seen different conclusions from prior works about whether the vision-language fusion should be integrated at early or late transformer layers. We conduct experiments to insert prompts at various layers. From Figure 5-right, we can see that inserting prompts into early layers (for example, layer 1–3 from bottom to top) achieves higher loss than inserting to late layers (for example, layer 1–3 from top to bottom). Thus in our case, prompts have greater significance at the late transformer layers. These results are also supported by the nature of the vision transformer hierarchy: lower layers mainly capture low-level fundamental visual details, while higher layers focus on high-level concepts that might be vital for downstream tasks.

In conclusion, we observe no single method that outperforms all the rest. For scenarios where a small number of parameters or datasize is available, we reckon that prompt tuning remains the preferred approach.

### 4.2 Flow matching policy evaluation

#### 4.2.1 Baseline studies

We compare our flow matching policy against: (i) Diffusion Policy (Chi et al., 2023) with DDPM and DDIM, (ii) Transformer-based behavior cloning, as customary in RVT (Goyal et al., 2023), RT-X (Padalkar et al., 2023), and (iii) Score-based generative models (Song Y. et al., 2020) in Section 4.4.

Note that we are aware of other competitive robot behavior cloning methods, including energy-based IBC (Florence et al., 2022), GAIL (Ho and Ermon, 2016), etc. Since extensive studies have been conducted and showed better performance of the diffusion policy against these methods, we choose the representative transformer and diffusion baselines for evaluation. Table 2 shows the hyperparameters used in flow matching and diffusion policy.

**TABLE 2:** Hyperparameters for flow matching and diffusion policy.

H-param	Ta	Tp	ObsRes	F-net	F-par	V-enc	V-par	Lr	WDe	Iters train (FM)	Iters train (DDPM)	Iters train (DDIM)	Iters eval
Activities of daily living	32	32	1 × 224 × 224	ConditionalUnet1D	72	ResNet-18	11	1e-4	1e-6	N/A	1/4/8/16	16	1/4/8/16
Push-T	8	16	1 × 96 × 96	ConditionalUnet1D	80	ResNet-18	11	1e-4	1e-6	N/A	1/4/8/16	16	1/4/8/16
Franka kitchen	8	16	1 × 60	ConditionalUnet1D	66	N/A	N/A	1e-4	1e-6	N/A	1/4/8/16	16	1/4/8/16
Robomimic	8	16	1 × 50	ConditionalUnet1D	66	N/A	N/A	1e-4	1e-6	N/A	1/4/8/16	16	1/4/8/16

Ta: action horizon. Tp: action prediction horizon. ObsRes: environment observation resolution. D-Net: diffusion/flow matching network. D-Par: diffusion/flow matching network number of parameters in millions. V-Enc: vision encoder. V-Par: vision encoder number of parameters in millions. Lr: learning rate. WDe: weight decay. Iters Train: number of training diffusion iterations. Iters Eval: number of inference iterations.

#### 4.2.2 Implementation details

Table 2 shows the hyperparameters used in flow matching and diffusion policy. All the baselines have been performed with an RTX 4090 GPU under the same conditions. We first train and test flow matching and baselines on our collected Real-world Activities of Daily Living (ADLs) dataset in a supervised manner with Mean Square Error Loss. In the following sections, we will also evaluate on other benchmarks (e.g., Push-T, Franka Kitchen, and Robomimic).

- ADLs benchmark: For 2D data, the training uses a RGB image with visual affordances as input, and the output is a trajectory in 2D pixel space. For the counterparts in 3D, the training takes the concatenation of RGB-D images with visual affordances as input, and outputs a trajectory in 3D Cartesian space. Since the ADLs task is not closed-loop, as shown in Table 3, we feed the image with all task-related object affordances to the flow matching policy, which directly outputs the entire trajectory. We randomly split the dataset with 80%–20% training and testing. The results are reported on the testing dataset, with 3-fold cross-validation, after 1, 000 epochs of training. For each baseline, all 10 tasks in our dataset are trained in a single policy in a supervised manner. To ensure fair comparisons, the corresponding hyperparameters across the flow matching policy and baseline methods are selected to remain consistent. Table 3 shows the task summary.

**TABLE 3:** Tasks summary.

Tasks	Rob	Obj	ActD	PH	Steps	Img	Closed-loop
Activities of daily living	1	≈30	3	8,000	N/A	**✓**	**✗**
Push-T	1	1	2	200	300	**✓**	**✓**
Franka kitchen	1	7	9	566	280	**✗**	**✓**
Robomimic	2	3	20	200	700	✗	✓

Rob: number of robots. Obj: number of objects. ActD: action dimension. PH: proficient-human demonstration. Steps: max number of rollout steps. Franka Kitchen and Robomimic involve 6D robot and gripper actions in the joint space. ADLs, and Push-T focus on robot end-effector trajectories. For clarity, we further explain that for our ADLs, tasks, as no closed-loop motion is considered here, we assume that the gripper closes when the first waypoint has arrived, and the low-level actions are executed between waypoints using a standard proportional-derivative (PD) controller.

#### 4.2.3 Main results

Table 4 presents the results of flow matching policy on our ADLs testing dataset for robot trajectory learning, comparing against baselines. We use two metrics for evaluation: (i) the average error of 2D and 3D trajectory estimation, and (ii) the average inference time. The trajectory error is averaged on each point of the trajectory.

**TABLE 4:** Results of flow matching policy against baselines and ablations.

Category	Methods	2D trajectory prediction (pixel) ↓	3D trajectory prediction (cm) ↓	Inference times (ms) ↓
Ours	Flow matching (transformer, 16-step)	1.061±0.103	1.233±0.128	140.71±7.672
	Flow matching (CNN, 16-step)	0.839±0.095	1.009±0.115	97.869±3.237
Ablations	Flow matching (CNN, 1-step)	0.889±0.090	1.031±0.082	13.737±0.558
	Flow matching (CNN, 4-step)	0.846±0.094	1.016±0.091	39.971±1.575
	Flow matching (CNN, 8-step)	0.841±0.099	1.011±0.097	78.361±0.303
Baselines	DDPM (1-step)	2.862±0.108	2.480±0.076	13.477±0.156
	DDPM (4-step)	0.889±0.101	2.415±0.088	47.284±0.926
	DDPM (8-step)	0.886±0.115	2.401±0.119	81.832±0.892
	DDPM (16-step)	0.884±0.209	2.397±0.124	97.727±0.156
	DDIM (1-step)	4.293±0.104	10.78±0.088	13.477±1.140
	DDIM (4-step)	0.876±0.073	2.303±0.094	41.536±5.274
	DDIM (8-step)	0.875±0.070	2.270±0.121	79.680±0.419
	DDIM (16-step)	0.873±0.071	2.265±0.123	105.583±6.705
	Transformer-based BC	2.813±0.207	4.935±0.158	7.594±0.505

We report the average error of 2D and 3D trajectory estimation and the inference time. Our flow matching method achieves the best trajectory estimation accuracy. We also investigate the effect of various design choices on flow matching performance, including network structures, training, and inference steps. Bold values indicate the best trajectory prediction accuracy across all methods and the inference time achieved by the proposed flow matching model.

Four observations could be made from this result:

- Generation quality: Flow matching (CNN-based, 16 steps) outperforms diffusion policy and Transformer baselines in terms of 2D and 3D trajectory prediction accuracy. The suboptimal performance of Transformer behavior cloning is expected as it is hindered by the nature of multi-modal action distribution, causing the averaging out across non-convex spaces.
- Stability: Figure 6 shows an example of training and testing loss of flow matching and diffusion policy with DDPM throughout the training process. We can see flow matching exhibits greater stability on both training and evaluation than diffusion policy.
- Inference time: We have two observations here: i) Table 4 showcases that flow matching with 16 steps achieves faster inference time compared to diffusion policy with 16 steps. We hypothesize that flow matching with linear pointwise flows generates straighter flows than DDPM and DDIM, and thus causes faster inference. ii) More importantly, Table 4 also showcases that 1-step flow matching (error: 1.031cm, time: 13.228 m) has achieved comparable performance as 16-step DDIM (error: 2.266cm, time: 98.680 m), but noticeably lowered inference time roughly by 85%. We hypothesize that this is because diffusion models solve a stochastic differential equation with a series of discrete steps to progressively refine the generated sample. Contrarily, flow matching trains continuously normalize flow models, leading to no significant improvements when increasing inference steps. Thus 2-step flow matching has achieved comparable performance as the 16-step diffusion policy, which considerably reduces the inference time for closed-loop robot manipulation. This is in line with the results obtained in the image generation domain. As pointed out by Stable Diffusion 3 (Esser et al., 2024), flow matching outperforms diffusion policy with fewer inference steps, making it particularly advantageous in scenarios that demand fast inference.
- Training resources: DDPM training and benchmarking demand significant resources for various training and inference steps. DDIM decouples the number of denoising iterations in training and inference, thereby allowing the algorithm to be trained one time with a large training iteration and use fewer iterations for inference to speed up the process. However, flow matching still achieves faster inference than DDIM.

#### 4.2.4 Ablations

We further ablate policy design choices.

Network Structure: As shown in Table 4, CNN-based flow matching achieves better results than transformer-based architecture. We hypothesize that Transformers might need additional hyperparameter tuning.

Trajectory Representation: We empirically test trajectory representation with 8, 16, 32 and 64 waypoints. More waypoints are not necessary, while fewer waypoints are unable to entirely encapsulate the complete long-horizon trajectories. We have found that, in general, the trajectory representation does not wield a significant influence on flow matching performance. The performance reported in the above main results section is achieved by using 32 waypoints.

### 4.3 Real-world robot evaluation

We deploy our flow matching policy on real robot manipulation for evaluation. We use a KINOVA Gen3 arm and an Azure Kinect camera for real-world robot experiments. Details can be found in the supplementary video.

We carry out 50 replications of trials for each baseline. The experimental protocol comprised 10 distinct tasks, each evaluated over five independent trials. Adhering to an open-loop testing framework on the Activities of Daily Living (ADL) dataset, any failure on the initial attempt was recorded as a categorical failure for that trial. We define task success based on three criteria: (1) the robot correctly grasps the target object, (2) the object/tool reaches the designated affordance region associated with the task, and (3) the robot performs the expected manipulation motion or achieves the intended object state change. A trial is considered successful when all three conditions are satisfied.

Our inference can run at approximately 30 Hz, (affordance learning: 18.340 m; flow matching with one-step inference: 13.228 m). Low-level components such as Inverse Kinematics and Task-Space Control could be operated on a dedicated, high-frequency thread in parallel with the policy inference in an asynchronous multi-threaded architecture. While the ADL scenarios in this work are primarily evaluated in an open-loop rollout for benchmarking, this low-latency profile demonstrates that our system is fundamentally capable of the high-bandwidth operation required for complex closed-loop manipulation tasks.

We have respectively compared the proposed flow matching policy against SoA baselines, including DDPM and Transformer policies. We also investigate whether our framework can seamlessly unify affordance learning and action generation by comparing against the end-to-end policy and off-the-shelf VLM-based trajectory generation.

Flow matching policy: From Table 5, we can see that flow matching outperforms DDPM and Transformer baselines.

**TABLE 5:** Real-world robot experimental results.

Methods (inference step)	Flow matching (16-step) ↑	Diffusion policy (16-step) ↑	Transformer BC ↑	Flow matching end-to-end ↑
Activities of daily living	0.82	0.76	0.44	0.74

Bold value indicates the highest success rate.

End-to-end learning (RGB images without affordances): To further investigate the benefit of using affordances and the capability of handling multimodal action distribution using our flow matching policy, we trained flow matching taking the raw RGB images and language tokens as input. This is similar to our proposed method but without the intermediate stage of affordance learning. It also involves some resemblance while not being entirely identical to the method in (Black et al., 2024) in an end-to-end learning manner. Interestingly, comprehensive examinations reinforce the argument that flow matching could handle multimodal action distribution. Figure 7 shows one example. From the left figure, we can see that when training a policy without affordances, the predicted trajectory (yellow) of moving the towel toward the trash for sweeping could be detached from the ground truth (red), but still a reasonable solution that allows for a successful robot execution. With affordance guidance, the prediction is closely aligned with the truth (Figure 7-right, Table). We also observe that for applications that demand higher precision in manipulation, like grasping the toothbrush handle, affordances offer greater guidance for shaping the manipulation policy.

**FIGURE 7:**
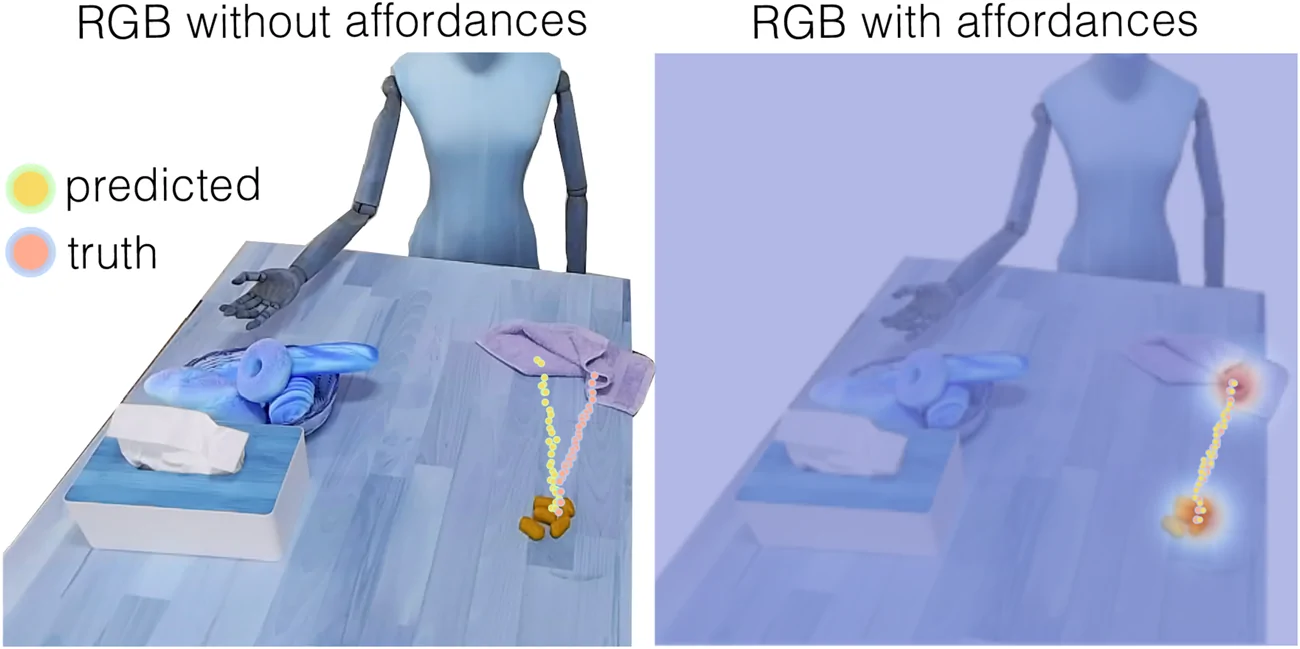
Ablations of using RGB images with/without affordances for policy training. Visual affordances guide the flow matching policy to generate a trajectory closely aligned with the truth. The policy without affordances might generate a trajectory detached from the ground truth, but still a reasonable solution. This reinforces the argument that flow matching policy could handle multimodal robot action distributions.

Off-the-shelf VLM-based trajectory generation: Our method uses an extra low-level module (flow matching) to learn a robot policy built on the high-level VLM reasoning. We have also conducted preliminary research to test another popular line of research, which uses a VLM to generate robot actions directly, as customary in the literature (Huang et al., 2023; Tanneberg et al., 2024). Consistent performance was only achievable through meticulous prompt design or fine-tuning. This result is expected, as several recent state-of-the-art research (e.g., HAMSTER (Li et al., 2025), π 0 (Black et al., 2024)) have shown that VLA models with robot learning modules outperform VLM-based trajectory generation.

### 4.4 Comparisons between flow matching and diffusion policy

To further investigate the performance of flow matching compared to diffusion policy, we benchmark the proposed methods on three more datasets, including closed-loop 6D robot actions and gripper actions: (i) Push-T (Florence et al., 2022), (ii) Franka Kitchen (Gupta et al., 2019), and (iii) Robomimic (Mandlekar et al., 2021).

- *Push-T* requires pushing a T-shaped block to a fixed target with a circular end-effector. Push-T takes RGB images with proprioception of end-effector location as inputs, and outputs end-effector actions in a closed-loop manner. The dataset includes 200 demonstrations.
- *Franka Kitchen* contains 7 objects for interaction and comes with a human demonstration dataset of 566 demonstrations, each completing 4 tasks in arbitrary order. The goal is to execute as many demonstrated tasks as possible, regardless of order. The training takes state-based inputs and outputs closed-loop robot joint actions and gripper actions.
- *Robomimic* consists of 5 tasks with a proficient human teleoperated demonstration dataset. We specifically focus on the transport task which includes 200 demonstrations. The policy takes state-based inputs and outputs closed-loop robot joint actions and gripper actions.

For each benchmark, the evaluation has been carried out across 500 different environment initial conditions, using the last checkpoint of each policy with 10 trials of replications. Thus, 5, 000 trials have been carried out in total per policy and benchmark. Variation is added on random initial conditions for the robot and object states. We respectively report the best and average performance in the 10 trials of replications of the last checkpoint. All state-based tasks are trained for 4, 500 epochs, and image-based tasks for 3, 000 epochs.

(Chi et al., 2023) has reported that setting prediction and action horizons greater than 1 helps the policy generate more consistent actions and compensate for idle portions of demonstrations; however, excessively long horizons can degrade performance due to slower reaction times. To ensure fair comparisons, we have selected the same corresponding hyperparameters across the flow matching policy and baseline methods to remain consistent. Table 2 shows the hyperparameters we have used in flow matching and diffusion policy. Table 3 shows the task summary.

Similar conclusions could be achieved from Table 6 and Figure 8, as in the Main Results section:

- Generation quality: The performances of flow matching and DDPM, DDIM, and the score-based model are comparable. We observe that score-based models can achieve higher peak performance, but their average performance tends to be less consistent.
- Inference time: Figure 8 shows how the number of inference steps affects the performance of flow matching and diffusion policy. We can observe that diffusion policy showcases better performance when applying more inference iterations with a trade-off of longer inference time, as it requires a series of discrete steps to progressively refine the generated sample (solid line). Score-based models with black-box numerical ODE samplers, as in the original research paper (Song Y. et al., 2020), need adaptive steps to achieve optimal results (around 100−300 ). Contrarily, flow matching has not shown significant improvements when increasing inference steps. From 8 steps onward, the performance of both flow matching and the diffusion policy increases only slightly with additional steps. However, the inference time (dotted line; we use frequency here) increases proportionally with the number of inference steps (dotted line). Therefore, flow matching considerably reduces the inference time for closed-loop robot manipulation. In the Push-T benchmark in Figure 8, 2-step flow matching (coverage: 0.8803, time: 13.098 m) has achieved comparable performance as a 16-step diffusion policy with DDIM (coverage: 0.8801, time: 98.268 m), but noticeably lower inference time, roughly by 86%.

**TABLE 6:** We present the robot evaluation performance in the format of (max performance)/(average of last checkpoint with 10 trials of replications), with each averaged across 500 different environment initial conditions.

Methods (16-step)	Push-T*^a^* ↑	Push-T*^b^* ↑	Franka kitchen ↑	Robomimic ↑
Flow matching	0.9035/0.7519	0.7363/ 0.6218	0.9960 /0.7425	0.9360/ 0.7289
DDPM	0.8840/0.7178	0.7360/0.6100	0.9840/0.6716	0.9359/0.7168
DDIM	0.8801/0.6372	0.7490/0.6167	0.9865/ 0.7471	0.9334/0.7073
Score-based	0.8950/0.6432	0.7659 /0.6186	0.9863/0.6892	0.9470 /0.4896

^a^ sampling range: [(50, 450), (50, 450), (200, 300), (200, 300), (−π,π) ].

^b^ sampling range: [(50, 450), (50, 450), (100, 400), (100, 400), (−π,π) ].

The metric used here is success rate, except for the Push-T, task, which uses target area coverage. We have used various sampling ranges (end-effector position, T-block position and orientation) for Push-T, environment initialization. For Robomimic benchmark, we specifically report results on the Transport task. Bold values indicate the best performance (highest maximum and average scores, respectively) across all evaluated methods.

**FIGURE 8:**
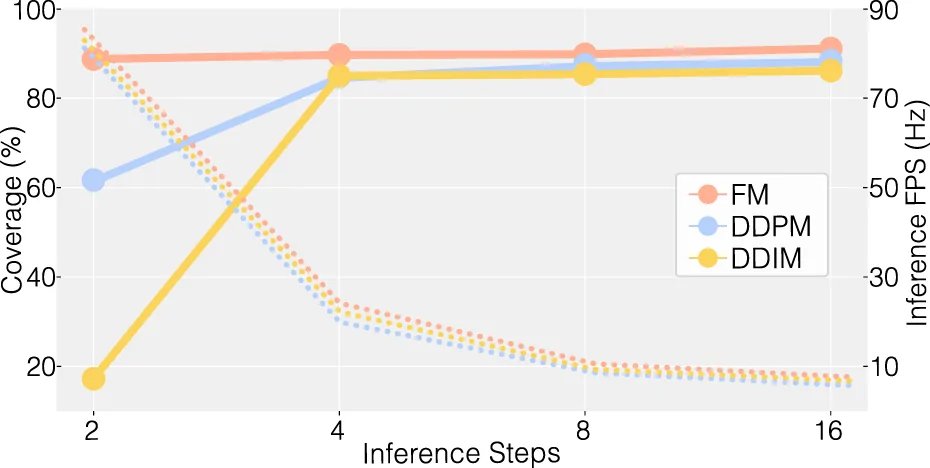
Generation quality (solid line) and inference time (dotted line, we use frequency here) comparison of flow matching and diffusion policy for varying values of inference steps.

Based on the above experimental observations, we conclude that flow matching formulates the robot policy generation problem as learning a deterministic or near-deterministic vector field (via an ordinary differential equation) that maps noise (or a simple prior) to target action distributions—this ODE-based framing avoids iterative stochastic denoising chains (as in diffusion policy) and yields fast, single- or few-step inference, which is crucial in closed-loop robotic settings. Besides, in manipulation tasks, the target action distribution is often multimodal (e.g., multiple possible grasp poses, multiple contact sequences) but still has strong structure (e.g., physics constraints, smooth trajectories through state-action space). Flow matching’s vector-field representation permits modeling such multimodality while preserving smooth interpolation and strong inductive bias towards consistent flows—thus potentially better capturing the structured trajectory manifold than, say, a GAN which may collapse modes, or a diffusion model which requires many steps. Moreover, because robotic manipulation is extremely sensitive to inference latency, stability, and feedback responsiveness, flow matching’s fewer-step generation is advantageous. The explicit mapping of a vector field lends itself more naturally to feedback correction and incorporation of geometric/physical structure (e.g., SE(3) equivariance) than purely stochastic models. Overall, flow matching combines fast inference and structured multimodal representation, making it a compelling choice for manipulation domains.

### 4.5 Failure analysis

Across the 10 real-world ADL tasks, performance varied based on task geometry and precision demands. Tasks with larger target areas and coarse spatial tolerances (e.g., passing the water to the human or sweeping the trash) proved easier, consistently achieving high success rates. Conversely, tasks demanding fine-grained manipulation precision, such as grasping a narrow toothbrush or fork handle, exhibited higher failure rates. Overall, most real-world failures stemmed from out-of-distribution (OOD) shifts, where variations in background appearance or significant camera view shifts. Increasing both the dataset size and diversity could help mitigate this issue. Unlike the simulation benchmarks, we performed open-loop manipulation in the real world, meaning that a failed first grasp attempt was counted as a failure; closed-loop manipulation could potentially address this limitation. Similar OOD bottlenecks were observed in simulation: in the Push-T benchmark, only successful trajectories were included during training, so the policy struggled with edge cases, such as a T-block stuck in a corner. Extending the imitation policy to an online or offline reinforcement learning framework may help address these limitations.

## 5 Discussion

In this section, we provide a detailed analysis of the advantages and limitations of our proposed affordance-based flow matching approach.

Inference time: The generation quality of flow matching and diffusion policy for robot manipulation are generally comparable. We would like to highlight that the focus of this work is not to outperform state-of-the-art general robot manipulation research. Instead, we have systematically studied the flow matching framework, which provides an alternative to diffusion policies for robot manipulation. We can not overlook the additional advantages of flow matching, including stable training, easy implementation, and most importantly, significantly faster inference with fewer inference steps than diffusion policy, suggesting forsaking the stochastic construction of the diffusion policy in favor of learning the probability path more directly as in flow matching.

We have evaluated how varying inference steps affect the performance of flow matching and diffusion policy. The original diffusion policy research (Chi et al., 2023) uses a large 100 training and inference steps with DDPM in their experiments. Based on our evaluation in Figure 8 and results from other research (Chisari et al., 2024), we can observe that beyond 8 steps, further increasing steps have only a marginal impact on the performance of diffusion policy, but with a trade-off of significantly longer inference time.

We are aware of recent research on accelerated diffusion techniques such as DPM-Solver (Lu et al., 2022), UniPC (Zhao et al., 2023), one-step diffusion policy with distillation (Prasad et al., 2024) and shortcut models (Frans et al., 2024). We primarily focus on conducting a comparative analysis of the fundamental structural differences between the flow matching and diffusion paradigms without specific post-hoc distillation or solver-side optimizations. Flow matching can also be enhanced through such techniques, attempting to compensate for high-curvature paths using higher-order numerical solvers, which introduces additional computational overhead per step. These specialized methods usually require significant training overhead, iterative reflow, or complex optimization schemes. Our goal is to demonstrate the computational tractability of the vanilla flow matching objective without the need for intensive distillation or multi-stage training.

Deterministic and stochastic tasks: Flow matching is inherently more deterministic than diffusion-based approaches, which can have implications in highly stochastic tasks—i.e., tasks where the same actions can lead to different outcomes due to environmental randomness or uncertainty, such as manipulating deformable objects, pushing objects on uneven surfaces, or navigating in dynamic environments. The impact of flow matching’s determinism also depends on the training dataset: with a diverse dataset covering a wide range of outcomes, flow matching can learn to predict the most likely trajectories effectively, whereas in limited or biased datasets, flow matching may fail to capture rare but plausible outcomes.

Trajectory smoothness: Although our flow matching framework effectively learns Cartesian-space or joint-space trajectory representations, it does not explicitly enforce second-order smoothness or torque feasibility during training or inference. In practice, the trajectories are executed through standard robot APIs (e.g., Kinova), which apply spline-based interpolation and internal velocity/acceleration limits to ensure dynamically feasible and smooth motion. Nevertheless, incorporating explicit smoothness or dynamic-feasibility constraints—such as velocity or acceleration regularization terms, or post-processing filters—could further improve motion consistency and physical plausibility. However, as noted in recent works (Römer et al., 2024; Zeng et al., 2025), these additions typically increase computational complexity, highlighting a trade-off between control fidelity and real-time efficiency. Exploring this direction is a valuable avenue for future research.

Transportation cost: While our method successfully leverages basic flow matching to generate robust and feasible affordance-based trajectories with a high success rate, we acknowledge that it does not explicitly pursue the minimal geometric transportation cost. More advanced flow techniques, such as Rectified Flow Matching (RFM) (Liu, 2022) or methods based on Optimal Transport (OT) (Villani, 2021), are mathematically designed to produce straight-line mappings that minimize this cost. However, implementing these methods introduces significant training and preprocessing overhead, often necessitating complex iterative or global optimization schemes. Given that our primary goal is to explore the fundamental structural efficacy and computational tractability and use affordances to shape the target distribution, we adopt the more efficient basic flow matching objective. We are deferring the complex, multi-objective optimization that simultaneously constrains geometric factors like path length and practical constraints to future work.

Scalability: While our parameter-efficient prompt tuning effectively leverages foundation models to learn manipulation affordances, current limitations present clear avenues for future research. First, our dataset was collected in a controlled environment with limited camera poses and lighting; demonstrating open-world generalization across domain shifts remains an ongoing challenge. Second, while our parameter-efficient prompt tuning leverages the semantic robustness of a pretrained foundation model to learn manipulation affordances, incorporating the spatial and semantic interactions between elements in multi-task scenarios, its ultimate performance against extreme linguistic noise is fundamentally data-driven. We plan to incorporate closed-loop real-world experiments, alongside expanding dataset diversity for greater open-world robustness. Furthermore, we are exploring extensions that integrate richer multimodal sensing—such as haptic, tactile, or force-torque feedback—with dynamics models to yield a more resilient, dynamics-aware affordance representation.

We have not explored applying flow matching for robot action generation in the reinforcement learning setting. Recent research (Zheng et al., 2023) has modeled a policy as a return-conditional flow matching model with classifier-free guidance to eliminate many of the complexities that come with traditional offline reinforcement learning and outperformed standard imitation learning. These methods are anticipated to be seamlessly adaptable to robotic manipulation scenarios; however, comprehensive validation through practical simulations and real-world experimentation remains imperative.

## 6 Conclusion

We have formulated a prompt tuning method for affordance map learning and flow matching policy for robot manipulation. The core idea of prompt tuning is to maximally exploit the pretrained foundation model, and rapidly excavate the relevance of foundation and downstream affordance learning tasks. We have proposed a flow matching policy constructing paths that allow faster inference and improved generation among robot behavior cloning methods. We qualitatively and quantitatively experiment on multiple robot manipulation benchmarks to prove that flow matching produces better trade-offs between computational cost and sample quality compared to prior competing diffusion-based methods.

## Data Availability

The raw data supporting the conclusions of this article will be made available by the authors, without undue reservation. Our code is publicly available here: https://github.com/HRI-EU/flow_matching.
